# Enhancing young people’s pregnancy loss and fertility awareness and knowledge via schools: a way forward

**DOI:** 10.1093/heapro/daae205

**Published:** 2025-01-17

**Authors:** Zara Harnett, Keelin O’Donoghue, Laura Linehan, Tamara Escañuela Sánchez, Rióna Cotter, Susan Dineen, Brendan Fitzgerald, Órla Power, Shelly Whelan, Holly Peters, Marita Hennessy

**Affiliations:** Pregnancy Loss Research Group, Department of Obstetrics and Gynaecology, University College Cork, Cork, Ireland; Pregnancy Loss Research Group, Department of Obstetrics and Gynaecology, University College Cork, Cork, Ireland; INFANT Research Centre, University College Cork, Cork, Ireland; Pregnancy Loss Research Group, Department of Obstetrics and Gynaecology, University College Cork, Cork, Ireland; Pregnancy Loss Research Group, Department of Obstetrics and Gynaecology, University College Cork, Cork, Ireland; National Perinatal Epidemiology Centre (NPEC), Department of Obstetrics and Gynaecology, University College Cork, Cork, Ireland; Pregnancy Loss Research Group, Department of Obstetrics and Gynaecology, University College Cork, Cork, Ireland; Pregnancy Loss Research Group, Department of Obstetrics and Gynaecology, University College Cork, Cork, Ireland; Department of Pathology, Cork University Hospital, Cork, Ireland; Pregnancy Loss Research Group, Department of Obstetrics and Gynaecology, University College Cork, Cork, Ireland; Department of Pathology, Cork University Hospital, Cork, Ireland; Pregnancy Loss Research Group, Department of Obstetrics and Gynaecology, University College Cork, Cork, Ireland; St Angela’s College, Cork, Ireland; St Angela’s College, Cork, Ireland; Pregnancy Loss Research Group, Department of Obstetrics and Gynaecology, University College Cork, Cork, Ireland; INFANT Research Centre, University College Cork, Cork, Ireland

**Keywords:** adolescent, education, health promotion, reproductive health, reproductive rights, reproductive physiological phenomena

## Abstract

Pregnancy and infant loss, in the form of miscarriage, stillbirth or early neonatal death, occurs in 20–25% of all pregnancies. Despite its prevalence and associated physical and psychological impacts, there remains a lack of public awareness and understanding of pregnancy loss, including amongst people of reproductive age. Drawing on evidence from a preliminary review of peer-reviewed and grey literature, we make the case for enhancing pregnancy loss and (in)fertility awareness, specifically focusing on young people in second-level education. We situate our work within reproductive health and reproductive justice frames, recognizing the impact of social factors on people’s reproductive lives, and the need for multi-level interventions to enable people to fully realize their reproductive rights and goals. Although schools provide an important setting to learn about and discuss topics relating to sexual and reproductive health—including pregnancy loss and fertility—current evidence suggests that this is not happening, despite young people’s desire to engage in such conversations. While there are barriers to addressing sexual and reproductive health issues within schools (including lack of access to teacher training, continuing professional development, appropriate and engaging resource material, teacher discomfort and lack of confidence), it is important that interventions are developed in collaboration with all relevant knowledge users, including young people themselves. This will help to ensure that any interventions developed are relevant, acceptable, feasible and effective. Further research is needed to explore how education around pregnancy loss and fertility can be best delivered within school settings.

**Contribution to Health Promotion**

- There is a lack of awareness of pregnancy loss and related issues amongst the general population and people of reproductive age.
- Sexual and reproductive health education targeted at young people tends to focus on abstinence, the prevention of unwanted pregnancies, contraception, abortion and sexually transmitted infections.
- Situating our work within reproductive health and reproductive justice frames, we emphasize the need to work with young people to develop independent, evidence-informed, developmentally appropriate interventions to enhance knowledge and awareness of pregnancy loss to enable people to fully realize their reproductive rights and goals.

## INTRODUCTION

In this perspective article, we make the case for enhancing pregnancy loss and fertility awareness, specifically focusing on young people in second-level education. We draw evidence from a preliminary review of peer-reviewed and grey literature, to inform our arguments and areas for future work. We situate our work within reproductive health and reproductive justice frames, recognizing the impact of social factors on people’s reproductive lives, and the need for multi-level interventions to enable people to fully realize their reproductive rights and goals.

*Reproductive health,* as defined by the United Nations (UN) and World Health Organization (WHO), is the ability for people to decide if and when they reproduce, as well as access to healthcare services that provide the best chance of having a healthy baby (see definition of terms in Table 1). This definition is based on the WHO’s concept of health, which emphasizes certain pre-requisites for health including economic resources, education and social justice, amongst others (WHO, 2021). *Reproductive justice* focuses on reproductive rights and social justice (Ross and Solinger, 2017). It recognizes the impact of social factors on women’s reproductive lives—including institutions, the environment, economics and culture—and the need to affect change at such levels rather than focusing on individual ‘choices’. The right to *not* have a child, the right to *have* a child, and the right to *parent* children in safe and healthy environments are the primary principles of reproductive justice (Ross and Solinger, 2017, p. 9). Pregnancy loss and fertility concerns fit within the first two of these principles. These topics also fit with the core components of reproductive justice, which include equal access to safe abortion, affordable contraceptives and comprehensive sex education (Ross and Solinger, 2017). As argued by bell hooks, a feminist focus on a broad range of reproductive health issues—not only abortion—is needed, across the life course, to advance the health and reproductive rights of all women (hooks, 2000). This needs to encompass a broad range of experiences—not merely the interests of privileged White women—and incorporate intersectionality (hooks, 2000). Furthermore, reproductive justice applies to everyone (Ross, 2017) and pregnancy loss and (in)fertility can affect a person regardless of their sex and/or gender identities. We therefore situate our call for action around enhancing fertility and pregnancy loss awareness and knowledge amongst young people within reproductive health and reproductive justice frameworks.

**Table 1:** Definition of terms

Term	Definition
Comprehensive sexuality education	‘… a curriculum-based process of teaching and learning about the cognitive, emotional, physical and social aspects of sexuality. It aims to equip children and young people with knowledge, skills, attitudes and values that will empower them to: realize their health, well-being and dignity; develop respectful social and sexual relationships; consider how their choices affect their own well-being and that of others; and, understand and ensure the protection of their rights throughout their lives’ (UNESCO *et al*., 2018, p. 16).
Fertility awareness	‘… the understanding of reproduction, fecundity, fecundability, and related individual risk factors (e.g. advanced age, sexual health factors such as sexually transmitted infections, and life style^1^ factors such as smoking, obesity) and non-individual risk factors (e.g. environmental and work place factors); including the awareness of societal and cultural factors affecting options to meet reproductive family planning, as well as family building needs’ (Zegers-Hochschild *et al*., 2017, p. 1793).
Relationships and sexuality education	‘… teaching and learning about the cognitive, emotional, physical and social aspects of relationships and sexuality. It aims to equip children and young people, in an age-appropriate manner, with the knowledge, skills, attitudes and values that will enable them to develop self-awareness and self-esteem; realize their health, well-being and dignity; develop positive and respectful, social and intimate relationships; consider how their choices affect their own well-being and that of others; and understand their rights and responsibilities in relation to themselves and others’ (NCCA, n.d.).
Reproductive health	‘…a state of complete physical, mental and social well-being and not merely the absence of disease or infirmity, in all matters relating to the reproductive system and to its functions and processes. Reproductive health therefore implies that people are able to have a satisfying and safe sex life and that they have the capability to reproduce and the freedom to decide if, when and how often to do so. Implicit in this last condition are the right of men and women to be informed and to have access to safe, effective, affordable and acceptable methods of family planning of their choice, as well as other methods of their choice for regulation of fertility which are not against the law, and the right of access to appropriate health-care services that will enable women to go safely through pregnancy and childbirth and provide couples with the best chance of having a healthy infant’ (UN Population Fund, 2014, p. 59).
Reproductive justice	… the ‘complete physical, mental, spiritual, political, social and economic wellbeing of women and girls, based on the full achievement and protection of women’s human rights’ (Ross and Solinger, 2017).
Reproductive rights	‘… rest on the recognition of the basic rights of all couples and individuals to decide freely and responsibly the number, spacing and timing of their children and to have the information and means to do so, and the right to attain the highest standard of sexual and reproductive health. It also includes the right to make decisions concerning reproduction free of discrimination, coercion and violence, as expressed in human rights documents’ (UN Population Fund, 2014, p. 60).
Social, Personal and Health Education	‘… a broad curriculum that helps children and young people learn about themselves and to develop and maintain healthy relationships. Over time, it also gives them the knowledge and skills to be able to make responsible decisions in relation to their overall health and wellbeing’ (NCCA Curriculum Online, 2024).

^1^Verbatim quotation. The authors of this perspective article do not support the use of the term ‘lifestyle’; see relevant critiques of this language and framing within the literature (Popay *et al*., 2010; Williams and Fullagar, 2019; Robinson and Smith, 2023).

In this article, we use the term ‘young people’ where possible. As defined by the WHO (WHO, 2024), the term encompasses people aged 10–24 years, including adolescents (10–19 years) and young adults (20–24 years). We avoid using ‘child’ or ‘children’ unless in the context of specific information sources (e.g. policies or research). Various terms are used to describe the context of teaching of sex education in schools, including ‘sex education’ and ‘relationships and sexuality education’ (RSE). Throughout this article we retain the terms used within the source document(s) to avoid any misinterpretation.

## WHY IS INCREASED AWARENESS OF PREGNANCY LOSS AND FERTILITY NEEDED?

Pregnancy loss and (in)fertility are linked in many ways and have many shared risk factors (Linehan *et al.*, 2021). For example, increased age—sometimes referred to in the context of ‘delayed childbearing’—is a risk factor for various forms of pregnancy loss including miscarriage (Quenby *et al*., 2021) and stillbirth (Lawn *et al*., 2016). It is also associated with an overall decline in fertility rates, more pronounced in women (Practice Committee of the American Society for Reproductive Medicine & Practice Committee of the Society for Reproductive Endocrinology and Infertility, 2022). Increased age is the single biggest risk factor for pregnancy loss and infertility (Andersen *et al*., 2000; Agenor and Bhattacharya, 2015). In this section, we will explore some of the research that highlights gaps in awareness and knowledge of pregnancy loss and (in)fertility amongst the general public, and also amongst people of reproductive age.

### Why focus on pregnancy loss?

Pregnancy and infant loss, in the form of miscarriage, ectopic pregnancy, molar pregnancy, stillbirth or early neonatal death, occurs in 20–25% of all pregnancies. Definitions of these terms can vary by country; generally agreed international definitions are outlined in Table 2. The majority of these losses—approximately 15% of clinically recognized pregnancies—are through miscarriage (Quenby *et al*., 2021). This includes second-trimester miscarriages, which occur in up to 3% of pregnancies (McNamee *et al*., 2014). The risk of later losses, in the form of stillbirth, is approximately 3.5 per 1000 total births (Blencowe *et al*., 2016). Though often discussed separately (i.e. not under the term ‘pregnancy loss’), pregnancies can also end in abortion or termination; involving approximately 39 per 1000 women aged 15–49 years (Bearak *et al*., 2020). In our work, we include abortion (also referred to as ‘termination of pregnancy’) within our definition of pregnancy loss. Shame and stigma pervade pregnancy loss, including between different types of losses (Hennessy and O’Donoghue, 2024). It is also important to state that pregnancy loss rates and experiences vary across a range of domains including geography (low- and middle-income and high-income countries) and socio-demographic factors, including ethnicity (Heazell *et al*., 2016; Lawn *et al*., 2016; Quenby *et al*., 2021) and sexuality and gender identity (Riggs *et al*., 2020; Croll *et al*., 2022). These are key considerations and challenges to address in efforts to raise knowledge and awareness to ultimately enhance care experiences and outcomes (Flenady *et al*., 2016; Hennessy and O’Donoghue, 2024).

**Table 2:** Definitions of various forms of pregnancy and infant loss

Term	Definition
Miscarriage	The loss of a pregnancy before viability (Quenby *et al*., 2021).
Ectopic pregnancy	When a fertilized egg implants itself outside of the uterus (womb) (Kelly-Harrington *et al*., 2024, p. 4).
Molar pregnancy	Occurs at the time of conception when the sperm and the egg join together and there is excessive development of the cells that form the placenta with little or no foetal (baby) development (Kelly-Harrington *et al.*, 2024, p. 4).
Stillbirth	A baby born following a foetal death at 154 days (22 + 0 completed weeks) or more of gestation (Blencowe *et al*., n.d.).
Early neonatal death	The death of a live born baby occurring within seven completed days of birth (Blencowe *et al*., n.d.).
Fatal foetal anomaly	A non-medical term used to describe anomalies that will lead to foetal or neonatal death; it is used interchangeably with the terms ‘lethal’ and ‘life-limiting’ (Jackson *et al*., 2023).

Various studies have highlighted deficits in knowledge about miscarriage, ectopic pregnancy, stillbirth and fatal foetal anomaly amongst the general public (Bardos *et al*., 2015; Nuzum *et al*., 2018; Power *et al*., 2018; Spillane *et al*., 2018; McCarthy *et al*., 2020), including university students (San Lazaro Campillo *et al*., 2018), and others who could benefit from such knowledge, including people with power to influence or determine policies and practices at local, regional, national or international level (Hennessy and O’Donoghue, 2024). Much research focused on lived experiences of pregnancy loss also highlights the lack of awareness of various forms of pregnancy loss and perceived susceptibility to experiencing such losses (Linehan, 2024; Hennessy *et al*., Forthcoming). Knowledge and awareness of second-trimester pregnancy loss (including miscarriage and termination for medical reasons) is also lacking, with people often unprepared for the realities of such losses, including the need to go through labour (Middlemiss, 2024).

Pregnancy advice, and ideals surrounding womanhood and motherhood, can negatively impact on women’s experiences, particularly when they experience pregnancy loss (Reagan, 2003; Murphy, 2019). This includes advice from government agencies and health professionals, amongst others, regarding engaging in certain health behaviours or following particular ‘rules’ during pregnancy (and even pre-conceptually)—such as not smoking, drinking or doing anything ‘wrong’—as a way to control pregnancy outcomes, or having a ‘mother’s instinct’ to know when something is wrong. This can result in feelings of blame, guilt and shame in the event of not engaging in particular behaviours or in cases of unexplained pregnancy loss (Reagan, 2003; Murphy, 2019). There have been various calls for interventions to address pregnancy loss-related knowledge gaps, including public awareness and health promotion programmes before and during pregnancy (de Bernis *et al*., 2016; San Lazaro Campillo *et al*., 2022; Escañuela Sánchez *et al.*, 2023), including within schools (Linehan, 2024; Hennessy *et al*., Forthcoming).

While it is not possible (nor, in some cases, desired on an individual level) to prevent all pregnancy losses, increased awareness and knowledge—coupled with structural interventions within healthcare and society at large—can contribute to prevention efforts, reducing and eliminating stigma, better equipping people for when pregnancy loss occurs, and improved psychological and social outcomes.

### Why focus on (in)fertility in the context of pregnancy loss?

Studies conducted across a range of countries and continents have demonstrated that people of reproductive age have inadequate fertility awareness (see definition in Table 1)—including of fertility, infertility risk factors and consequences of increased time to pregnancy or ‘delayed childbearing’ (Pedro *et al*., 2018; Ren *et al*., 2023; Linehan, 2024), as well as the combined experiences of infertility and miscarriage (Linehan, 2024). For example, a systematic review undertaken to explore the knowledge around fertility awareness and its associated factors found that in several studies, on average, fewer than half of the people knew the definition of infertility (Pedro *et al*., 2018). Another systematic review found that knowledge of age-related fertility decline is poor (García *et al*., 2018). Such lack of knowledge around fertility is said to be a ‘factor in the failure to achieve parenting goals’ with having adequate knowledge offering the ability to make informed decisions related to reproductive health (Ren *et al*., 2023, p. 1). While the limited number of interventions to increase fertility knowledge that have been conducted to date show promise, further research is needed to enhance the evidence base regarding intervention effectiveness, particularly around interventions targeting young people in school (García *et al*., 2018). Martins and colleagues have proposed a conceptual framework for fertility education that includes a focus on children and adolescents, with integration within the curriculum advocated (Martins *et al*., 2024).

## THE INVISIBILITY OF PREGNANCY LOSS AND FERTILITY-RELATED EDUCATION WITHIN SCHOOLS

Access to sexual and reproductive health education underpins many international policies and is where pregnancy loss and fertility-related topics can potentially be addressed. In its *2030 Agenda for Sustainable Development*, the UN presented several goals, one of which centred on universal access to ‘sexual and reproductive health-care services, including for family planning, information and education, and the integration of reproductive health into national strategies and programmes’ (UN, 2015, p. 16). The UN Convention on the Rights of Children further recognizes a child’s right to have access to ‘comprehensive and inclusive sexual and reproductive health education based on scientific evidence’ (UN, 2016). The availability of comprehensive sexual education (see definition, Table 1); however, varies internationally (UNESCO, 2021).

From our review of the literature, we observed that little research has been conducted internationally on education around pregnancy loss and fertility within second-level education or targeted at young people aged 12–18 years. This is mirrored in reviews of the international curricula, which note that sexual and reproductive health education aimed at young people thus far has tended to focus on abstinence, the prevention of unwanted pregnancies, contraception, abortion and sexually transmitted infections (Nargund, 2015). Indeed, similar has been noted in relation to social marketing-based sexual health interventions for young people (Putkonen *et al*., 2024). Furthermore, available research demonstrates the lack of coverage of pregnancy loss and fertility-related topics within curricula and the expressed need for such gaps to be addressed. For example, in their synthesis of qualitative studies, Pound *et al.* found that young people (in various countries, including the UK and Ireland) highlighted that insufficient time was given to topics such as the pros and cons of contraception, pregnancy options such as adoption, abortion, teenage pregnancy and unbiased information with regard to abortion and how to deal with an abortion (Pound *et al*., 2016). These findings are echoed in a study conducted in the UK, which found that key topics such as fertility, preconception health, pregnancy, miscarriage, infertility (except in the context of artificial reproductive technologies) were missing from school curricula for 14–18-year-olds (Maslowski *et al*., 2023). Fertility-related topics, including endometriosis and polycystic ovarian syndrome (PCOS) were also absent in the curricula examined (Maslowski *et al*., 2023). Within the aforementioned study, young people wanted topics relating to fertility issues included in the curriculum, as well as topics relating to labour and delivery, postpartum, and women’s health in general. These findings were mirrored somewhat in a survey of 16- to 18-year-old school students in England: while the majority had learnt about topics such as sexually transmitted infections, contraception, puberty and the menstrual cycle in school, topics such as abortion, miscarriage, menopause, endometriosis and PCOS were more likely to be learnt about outside of school—the most common sources of sex education in this context being the internet and social media (Maslowski *et al*., 2024b). Misunderstanding of some reproductive health topics was evident, including the maximum age at which women are able to conceive a child naturally. Students stated that they would welcome a broader focus within RSE, with topics such as fertility and infertility, miscarriage, abortion, and how to access sexual and reproductive health services, amongst others, suggested for addition to the curriculum (Maslowski *et al*., 2024b).

Over half of students in a survey of the knowledge of secondary school pupils in Belgium aged 15–19 years noted the lack of discussions about infertility, IVF and egg freezing topics in school; most wanted more information on reproductive issues, with some stating their preference for a dedicated weekly course (Delbaere *et al*., 2024). Based on their survey research and focus groups with students and teachers, Delbaere and colleagues developed an online platform (www.allesovervruchtbaarheid.be/en) to enhance fertility awareness and education (Delbaere *et al*., 2024). Recognizing that approximately 10–24% of pregnancies end in miscarriage, Maslowski *et al.* (Maslowski *et al*., 2024a, p. 715) argue that sex education, ‘is not adequately preparing young adults, especially females, for challenges they might face during their adult lives’, in England specifically, though this could be extended to other countries based on findings outlined above.

In our review of the literature, we also identified other interventions that aim to enhance fertility knowledge amongst young people, though not limited to within schools. For example, in 2019, the industry-funded Fertility Education Initiative, a specialist interest group of the British Fertility Society—launched an educational animation series aimed at enhancing fertility awareness and knowledge amongst young people, not restricted to school settings (British Fertility Society, 2019, 2024). In June 2024, the International Reproductive Health Education Collaboration (IRHEC; which also has industry links) launched an educational poster—co-designed with young people from Denmark, Sweden and the UK—to improve young people’s knowledge about fertility (Harper, 2024; IRHEC, 2024), but this does not address pregnancy loss beyond highlighting the increased risk of miscarriage when male partners are aged over 40. Fertility Europe—an umbrella body for patient associations that receives industry funding (Fertility Europe, n.d.)—also developed an interactive quiz/game called ‘FActs!’ to enhance fertility awareness (Fertility Europe, 2024). There is only one mention of pregnancy loss within the quiz, relating to the impact of alcohol intake by women on early pregnancy loss. It could be claimed that the focus of these organizations and/or resources is fertility and therefore attending to pregnancy loss is, or should not be, within their remit. We would suggest, however, that there is a need to address pregnancy loss alongside fertility awareness, given the shared risk factors (discussed previously) and adverse outcomes associated with both, including pregnancy loss (in addition to unsuccessful transfers) in the case of artificial reproductive technologies (Linehan *et al*., 2021).

## HOW CAN WE ENHANCE EDUCATION AROUND PREGNANCY LOSS AND (IN)FERTILITY?

In this section, we explore how education around pregnancy loss and (in)fertility for young people can be enhanced, including the role of schools and teachers, factors that influence the teaching of reproductive and sexual health-related topics in schools (where pregnancy loss and (in)fertility-related topics can be addressed), and additional factors to consider when developing and implementing interventions. Schools are important settings for promoting the health and well-being of young people (WHO and UNESCO, 2021). If young people do not learn about reproductive health-related issues in school, they may learn about these elsewhere; for example, from the internet or social media, as noted in the study by Maslowski and colleagues (Maslowski *et al.*, 2024b). The quality of online information about pregnancy loss is variable, however, with key information often omitted and/or inaccessible (Ehrenreich *et al*., 2019; Escañuela Sánchez *et al*., 2020; Linehan *et al*., 2023). Online misinformation about reproductive health issues—including inaccurate or exaggerated risks regarding abortion, or exaggerated claims of the efficacy of fertility-related interventions—can result in beliefs and behaviours or courses of action, which may result in adverse, or at the very least unanticipated, outcomes (Pagoto *et al*., 2023; John *et al*., 2024). Access to accurate information is an important aspect of achieving reproductive health and reproductive justice (Ross and Solinger, 2017). Thus, school-based interventions addressing knowledge and awareness of pregnancy loss are worthy of further exploration.

### The role of schools/teachers in sexual and reproductive health education

Schools have been highlighted as ‘well placed’ to facilitate discussions around important topics relating to sexual health education (Mitchell *et al*., 2020, p. 1), including pregnancy loss and fertility-related topics. Teachers play an ‘integral role’ in providing comprehensive sexuality education (see definition in Table 1) to young people, with the qualities a teacher brings to the classroom environment found to be significantly related to an increase in knowledge around health education, including sexual health (Costello *et al*., 2022, p. 13). Given the significant role that school plays in children and young people’s lives, it provides an optimal environment within which to provide students with information and education relating to pregnancy loss and fertility. Notably, however, the adequacy of existing curricula in this regard has been questioned, as previously outlined.

We provide details on the context to the teaching of RSE in Ireland in Box 1. The information presented is based on our analysis of national curriculum guidelines and associated documentation to support their implementation, including reports, a Junior Cycle Social, Personal and Health Education (SPHE) textbook (we were unable to source a Senior Cycle textbook, which is perhaps reflective of the attention in this area), and sample timetables obtained from several secondary schools across Ireland. It aims to be illustrative of what happens in practice rather than a systematically conducted, in-depth documentary analysis. We acknowledge that different terms are used to describe the teaching of sex and reproductive health issues in a school environment within the literature, including comprehensive sexuality education (CSE), SPHE and RSE, and that these terms vary between countries.

Box 1. **THE TEACHING OF RSE IN IRELAND**

**BACKGROUND**

In 2016, the UN Committee on the Rights of the Child highlighted the ‘severe lack of access to sexual and reproductive health education’ for adolescents in Ireland (UN, 2016, p.13). Three years later, the Department of Education and Skills in Ireland recognized sexuality education as a human right (Houses of the Oireachtas, 2019).

RSE in Ireland is mandatory. This means that all students in secondary school—both Junior and Senior Cycle (young people aged 12–16 and 15–18 years, respectively)—should receive RSE within their school timetable, as part of SPHE. Schools are required to provide a minimum of six RSE lessons per year in senior cycle (indeed each year from junior infants (age 4/5) upwards); however, as we will later discuss, most schools—approximately four in five—are not timetabling SPHE (NCCA, 2022).

SPHE has been recognized as a crucial safe space, providing students with a supportive, non-judgmental environment whereby they are able to and encouraged to engage in dialogue and self-reflection (HSE, Health and Wellbeing, 2023). It is ‘grounded in an approach that is holistic, student-centered, inclusive, and age and developmentally appropriate’ (NCCA, 2023, p. 8). Thus, it provides an ideal opportunity to explore issues relating to pregnancy loss and (in)fertility.

**TOPICS COVERED WITHIN RSE CURRICULA**

The curriculum framework is not prescriptive, therefore, what is taught within the classroom is at the teacher’s discretion. This can significantly impact the education provided to young people on topics that are relevant to their futures regarding education around sexuality, relationships and reproductive health. This is highlighted in numerous published guidelines and documents that we reviewed.

For example, in a document developed by Health Service Executive (HSE) and supported by the Department of Education and the NCCA titled *‘Relationships and Sexuality Education Year 2*’, it presents activities as guidelines, noting that teachers are ‘best placed to decide’ on what will be most effective within their classroom environment (HSE, Health and Wellbeing, 2023, p. 2). Encouragingly, within the document—developed for RSE in Junior Cycle—there is reference to factors influencing fertility such as age, health and hormonal or immune conditions (including discussion of female and male risk factors) and suggested discussion points (HSE, Health and Wellbeing, 2023). The topic guidelines covered in Junior Cycle are expanded upon in a draft document for consultation for SPHE in senior cycle (NCCA, 2023). The exploration of sexual and reproductive health, including fertility and responses to unplanned pregnancy, is a learning outcome under Strand 2 (2.9). The document stresses that schools are encouraged to be flexible with the framework and to plan SPHE in relation to their unique setting. Teachers are encouraged to select what modules to cover based on what is important and of interest to students. While guided by the curriculum, teachers can focus more on relationships rather than sexuality content, as what is delivered is at their discretion.

From our examination of documents pertaining to the implementation of the national curriculum guidelines, we observed an under representation of SPHE provision. We found little evidence of development with regard to the inclusion of topics such as fertility and pregnancy loss in the classroom setting. For example, exploration of the current curriculum and examination of a Junior Cycle textbook (published in 2017, though still in use), revealed that the content—both the diagrams and the dedication of only three pages within the book to discuss sexual health—is outdated and insufficient (Potts and O’Grady, 2017; HSE, Health and Wellbeing, 2023).

**GAPS IN YOUNG PEOPLE’S REPRODUCTIVE AND SEXUAL HEALTH EDUCATION IN IRELAND**

Our analysis demonstrates important progression of the curriculum to include topics such as practicing safe sex, exploring thoughts, values, attitudes and feelings about relationships, and understanding sexuality and sexual orientation (NCCA, 2011). However, it remains limited regarding other essential topics relating to reproductive health, including—but is not limited to—fertility and pregnancy loss.

### Factors that influence the teaching of reproductive and sexual health-related topics in schools

Provision of a full range of sexual health and well-being topics, scaffolded across ages with developmentally appropriate content and teaching, embedded in supportive school environments and across subject areas, has the potential to improve sexual, social, and emotional health and academic outcomes for young people (Goldfarb and Lieberman, 2021). Various factors have been identified as influencing—positively or negatively—the provision of reproductive and sexual health in schools, including both the breadth and depth of topics covered. In their qualitative systematic review of teachers’ perspectives on providing sexual and reproductive health education in primary and secondary schools, Walker *et al.* (Walker *et al*., 2021) found that adequacy was linked to teachers’ confidence (including factors such as their perceived role, training, experience, relationship with students and access to resources), school policies and environment (including teaching what is mandated, administrative support, advocacy, school culture/ethos, parents, breadth of topics taught, different teaching approaches), and the priority given to such education (including advocacy, available/allotted time and resources, external providers, access to healthcare and care taken to teach sexual and reproductive health topics). Included studies were from a range of high-income countries including Australia, Canada, Ireland, Japan, the UK and the USA.

Teachers require appropriate knowledge, expertize, confidence and appropriate pedagogical skills to teach RSE (Lodge *et al*., 2024). However, deficits in initial teacher training at undergraduate or postgraduate levels regarding specialist knowledge in SPHE/RSE have been identified (O’Brien *et al*., 2021; Lodge *et al*., 2024), as well as lack of access to continuing professional development activities, lack of qualification in SPHE/RSE, the absence of mandated curriculum content and engaging, appropriate and up-to-date resources, and fear of external criticism, especially from parents, coupled with the lack of time allowed for the RSE in many schools’ timetables (Keating *et al.*, 2018; Lodge *et al*., 2024). Inadequate timetabling is certainly an issue. For example, in Ireland only 22% of 690 schools offer Transition Year timetabled SPHE classes; this decreases to 18% in 5th year (~16/17 year olds) and 17.5% in 6th year (~17–18 year olds) (National Council for Curriculum and Assessment [NCCA], 2022). Sex education is also a particularly politicized area of the curriculum and frequently subject to legal challenges, which poses a barrier to its teaching (Bourke *et al*., 2022). Discomfort around certain topics poses a significant risk to the lack of education around certain topics within RSE, particularly sexuality (Mayock *et al*., 2007); this can arise due to various factors including a lack of training or inability to discuss topics in a non-judgemental way (Pound *et al*., 2017; Costello *et al*., 2022). It can also be impacted by the religious ethos within schools. For example, in Ireland, half of post-primary school enrolments in 2023 were in schools that had a Catholic (47.8%) or a Church of Ireland (3.0%) ethos (Department of Education, 2023). In their study, Mayock *et al*. (Mayock *et al*., 2007) noted the ambiguity around the impact of religious ethos on RSE in Ireland, which led to differences in how teachers approach the content of RSE—particularly contraception, condom use and homosexuality—with some acknowledging the school’s ethos by way of preface to such discussions, while others discussed such topics on a one-to-one basis with students. One further point to acknowledge within this section. Teachers may also have lived experiences of pregnancy loss and/or infertility (Dunn, 2023; Kelly-Harrington *et al*., 2024), which may—or may not—influence their motivation or capability to teach around these topics. This requires consideration in the development and implementation of any interventions and research with teachers to examine their views and experiences.

Thus, we contend that equipping teachers with the necessary knowledge and skills, supportive environments and structures (including policies) and prioritizing the teaching of sexual and reproductive health issues is important when seeking to enhance pregnancy loss and fertility awareness amongst young people in schools.

## THE WAY FORWARD: WHAT IS NEEDED

In addition to the factors previously outlined, other aspects to consider in the development and implementation of interventions include the need for independent, evidence-based interventions, appropriate communications and messaging and working with young people to develop interventions that meet their needs.

### Independent, evidence-informed interventions

The European Expert Group on Sexuality Education has highlighted the importance of providing scientifically accurate, non-judgemental, age-appropriate sexual education (Maunsell *et al*., 2021). It is important that pregnancy loss and (in)fertility education efforts are evidence-informed, and free of industry influence (e.g. through inappropriate partnerships and/or sponsorship/funding), given the potential for corporations to act or engage in ways or practices that undermine women’s health while claiming to be advancing gender equity or female empowerment agendas (McCarthy *et al*., 2023). Relevant to our focus, corporations can be served by individual responsibility narratives, which open up markets for products and services to optimize pregnancy outcomes, while they also engage in PR efforts—for example, funding pregnancy loss awareness campaigns or activities (Browne, 2024) or promoting their own pregnancy loss-related leave policies.

Further research is also needed to enhance the development and implementation, and ultimately effectiveness, of sexual and reproductive health interventions delivered within schools. In their review of reviews, including studies from low- and middle-income and high-income countries, Denford *et al.* (Denford *et al*., 2017) found inconsistent findings for impacts on sexual health behaviours but noted that comprehensive (CSE) interventions were more effective in improving knowledge, attitudes, behaviours and health-relevant outcomes when compared with other intervention types, for example, abstinence-only. More recently, Niland et al. (Niland *et al*., 2024) conducted a systematic review on the role of school-based sex education interventions, examining what behaviour change techniques (BCTs or ‘active ingredients’) were associated with intervention effectiveness. Five BCTs appeared more frequently within effective interventions: information about health consequences; information about social and emotional consequences; demonstration of the behaviour; behavioural practice/rehearsal; instructions on how to perform the behaviour. These could be examined in any further interventions regarding enhancing awareness and knowledge of pregnancy loss and (in)fertility. Limitations in length of follow-up reported within studies should be noted (Denford *et al*., 2017; Niland *et al*., 2024) and appropriate outcomes targeted in any future interventions, particularly given the longer-term outcomes surrounding pregnancy/pregnancy loss.

### Appropriate communication and messaging

Enhancing health literacy—including knowledge about fertility, contraception, safe sexual practices and factors that influence pregnancy outcomes—is one component of efforts to advance reproductive health (Kilfoyle *et al*., 2016). However, in our efforts to raise awareness and break the silence around topics such as miscarriage, we need to ‘connect miscarriage as a feminist issue with wider struggles for social justice’ and do more rather than merely talk more (Browne, 2024, p. 3). Personal responsibility narratives further stigma surrounding pregnancy loss (Browne, 2024). We must also resist the temptation of narratives that support ‘White nationalist pronatalist “birth rate” panics and a profit-driven pregnancy industry whose expansion depends on amplifying the cultural celebration of “successful” pregnancy as having-a-baby’ rather than acknowledging all pregnancies, however they end (Browne, 2024, p. 9). It is also important to acknowledge that not everyone wishes to have children. In a survey of parenthood intentions for 16–18-year-old students, 36% indicated that they did not want to have children in the future or were unsure, citing concerns around pregnancy and childbirth (females), their ability to be ‘good’ parents, and the current state of the world, amongst others (Biswakarma *et al*., 2024). Many students had also concerns about the possibility of having children in the future (45%), including fears or worries surrounding pregnancy, childbirth and their fertility and their ability to have ‘healthy’ children (Biswakarma *et al*., 2024).

During pregnancy, and even when contemplating pregnancy, women are expected to engage in a range of ‘healthy’ behaviours—monitoring and controlling their bodies—to ensure the health and well-being of their foetuses/babies, and bearing the full responsibility for this (Lupton, 2012; Ross, 2016). This can create anxiety and self-blame for women (Ross, 2016). It also now extends to ‘preconception health’ with the imperative for women of reproductive (or ‘childbearing’) age to prepare for a ‘healthy pregnancy’ as part of ‘a new focus on improving health *before* conception’, whether pregnancies are intended or not (Stephenson *et al*., 2021, p. 233). ‘Privatizing risk’ to the body of the pregnant woman ignores the socio-cultural, political and commercial determinants of health and contexts within which health-related decisions are made (Lupton, 2012, p. 338). Furthermore, reproductive justice recognizes the biological and social nature of reproduction and how the context in which people become parents or not cannot be ascribed to individual choice (Ross and Solinger, 2017). Efforts to address knowledge and awareness of fertility and pregnancy loss need to avoid placing responsibility for ‘successful pregnancies’ solely on women (Sharp *et al*., 2018, 2019), and indeed individuals more generally.

Any interventions addressing education and awareness—with young people, or broader population groups—need to adopt a critical approach, within reproductive health and reproductive justice frameworks. Risk communication needs to be evidence-informed at multiple levels, including what is communicated, how, and by whom. For example, the use of fear appeals or fear arousal to change awareness and risk perception is not effective, but may be useful when combined with messages that enhance self-efficacy and people’s ability to change their behaviour (Kok *et al*., 2018). The acceptability and effectiveness of peer-led approaches should also be explored as currently the evidence base regarding peer-led health interventions in schools lacks clarity and quality overall resulting in mixed findings (Dodd *et al*., 2022). Guest speakers and ‘experience experts’ in RSE can be viewed positively by young people also (Cense *et al*., 2020).

As discussed above, there are gaps in young people’s fertility knowledge and, while they are open to learning more about fertility, how this knowledge—and associated risks and implications for their (current and future) lives—is communicated needs careful consideration (Boivin *et al*., 2019). Research is needed to identify what and how to communicate fertility-related messaging to young people of different ages (Boivin *et al*., 2019); similar is required for pregnancy loss-related messaging.

### Working with young people to develop interventions that meet their needs

Research has emphasized the importance of including the views, knowledge, experiences and actions of people targeted by interventions when developing them, including young people (Larsson *et al*., 2018). This includes seeking children’s thoughts and opinions regarding the ways of teaching that influence the effectiveness of RSE in primary schools (Aguilar Alonso *et al*., 2024), and co-producing RSE curricula and resources with young people (Martin *et al*., 2020; Scott *et al*., 2020; Bourke *et al*., 2024). It is also important to involve other interest holders—for example, parents/caregivers, teachers, people with lived experience and others—in the design and implementation of interventions (Martins *et al*., 2024). Partnership between parents and schools is essential for the development and implementation of comprehensive and culturally sensitive and comprehensive school-based sexuality education curricula (Morin and Marwah, 2024). The importance of recognizing young peoples’ role in taking the lead in the designing of sexuality programmes has been highlighted by Allen (Allen, 2005), with young people further described by Coll and colleagues (Coll *et al.*, 2018, p. 158) as ‘creative educational stakeholders’. It is therefore important to not simply see young people as participants or recipients but as important partners in the development and implementation of interventions that can in fact be more ‘effective and efficient’ (Larsson *et al.*, 2018, p. 2). Meaningfully involving young people in activities to improve sexual and reproductive health, including pregnancy loss and fertility awareness, is necessary. It requires involving them from the outset, developing conditions and trusting relationships to support the involvement of diverse groups, creating different ways for them to contribute, and formatively evaluating young people’s experiences of involvement (Lewis *et al*., 2023).

## CONCLUSION

In this article, we set out to discuss the importance of increasing awareness and offering young people the opportunity to learn about pregnancy loss and fertility. We presented evidence that outlines the lack of knowledge of pregnancy loss and fertility awareness among the general public, especially people of reproductive age. Schools provide an important environment for the facilitation of discussions of topics relating to sexual health education, including pregnancy loss and (in)fertility; however, current evidence would suggest that this is not happening. Young people themselves acknowledge their lack of knowledge around these important topics despite their desire to engage in conversations around them. People with lived experience of pregnancy loss have also suggested that such topics should be discussed within schools to better prepare people for when pregnancy loss happens. One way to provide this opportunity is to expand the current discussions that are occurring within SPHE/RSE and potentially other curricular areas in schools that may vary by country/region. While there are barriers to addressing sexual and reproductive health issues within schools, it is important that interventions are developed in collaboration with all relevant knowledge users, including young people themselves. This will help to ensure that any interventions developed are relevant, acceptable, feasible and effective. Further research is needed to explore how education around pregnancy loss and fertility can be best delivered within school settings.

Finally, it is important to acknowledge that activities to increase knowledge and awareness are only one component of what is needed to ensure reproductive justice for all. Efforts to address socio-economic and structural inequalities and injustices must be pursued at pace also. It is important that any educational activities address such broader determinants, enabling young people to learn about, and potentially advocate around, them. While there may be challenges encountered in addressing pregnancy loss and fertility awareness in schools, providing young people with information around these issues that is accurate, relevant and developmentally/age-appropriate will equip them with important knowledge and skills to realize their reproductive rights and goals.

## Data Availability

No new data were generated or analysed in support of this research.
